# Parents, but not their children, demonstrate greater delay discounting with resource scarcity

**DOI:** 10.1186/s12889-023-16832-z

**Published:** 2023-10-12

**Authors:** Alyssa M. Button, Rocco A. Paluch, Kenneth B. Schechtman, Denise E. Wilfley, Nancy Geller, Teresa Quattrin, Stephen R. Cook, Ihouma U. Eneli, Leonard H. Epstein

**Affiliations:** 1 Division of Population and Public Health Science, Pennington Biomedical Research Center, Baton Rouge, LA USA; 2 Department of Pediatrics, Jacobs School of Medicine and Biomedical Sciences, University at Buffalo, 3435 Main Street, Building #26, Buffalo, NY 14214 USA; 3 Department of Psychiatry, Washington University in St. Louis School of Medicine, St. Louis, MO USA; 4 National Heart, Lung, and Blood Institute, National Institutes of Health, Bethesda, MD USA; 5 Department of Pediatrics, University of Rochester Medical Center, Rochester, NY USA; 6 Department of Pediatrics, Nationwide Children’s Hospital, Columbus, OH USA

**Keywords:** Delay discounting, Scarcity, Obesity, Socioeconomic status

## Abstract

**Background:**

Individuals with obesity tend to discount the future (delay discounting), focusing on immediate gratification. Delay discounting is reliably related to indicators of economic scarcity (i.e., insufficient resources), including lower income and decreased educational attainment in adults. It is unclear whether the impact of these factors experienced by parents also influence child delay discounting between the ages of 8 and 12-years in families with obesity.

**Methods:**

The relationship between indices of family income and delay discounting was studied in 452 families with parents and 6–12-year-old children with obesity. Differences in the relationships between parent economic, educational and Medicaid status, and parent and child delay discounting were tested.

**Results:**

Results showed lower parent income (*p* = 0.019) and Medicaid status (*p* = 0.021) were differentially related to greater parent but not child delay discounting among systematic responders.

**Conclusions:**

These data suggest differences in how indicators of scarcity influence delay discounting for parents and children, indicating that adults with scarce resources may be shaped to focus on immediate needs instead of long-term goals. It is possible that parents can reduce the impact of economic scarcity on their children during preadolescent years. These findings suggest a need for policy change to alleviate the burden of scarce conditions and intervention to modify delay discounting rate and to improve health-related choices and to address weight disparities.

## Introduction

Delay discounting, or the tendency to choose small immediate over larger but delayed rewards, is a measure of a person’s temporal window that indicates a preference for immediate gratification [1]. It is a process whereby a reduction in the value of a reinforcer is a function of the delay to its receipt [1–3]. It is a trans-disease process [4, 5] that is related to obesity and health maintenance behaviors [3, 6–9]. Delay discounting rates can be increased by genetic, neural, environmental, and other factors and modulates the risk of other disorders (e.g., obesity, diabetes) through maladaptive health behaviors (e.g., fast food consumption, poor medication adherence) [10]. The tendency to discount the future and choose present rewards is also related to many maladaptive behaviors and behavioral disorders such as internet gaming, gambling, cigarette use, substance abuse, medical treatment adherence, and risky sexual behaviors [11–16]. Delay discounting is related to health because people who discount the future do not engage in behaviors that may promote health in favor of immediate gratification [15, 17].

Delay discounting is relevant for weight control or the prevention of weight gain which requires a person to engage in healthy behavior now for future benefits. Food is a powerful primary reinforcer [18], and people with obesity may choose hyper-palatable, highly reinforcing foods [19] rather than avoiding these foods for the distal benefits of a healthy weight [3]. Similarly, people with obesity find sedentary behaviors more reinforcing than more physically active behaviors, and they must choose to reduce time spent in pleasurable sedentary behaviors and engage in less reinforcing physical activity to gain future benefits based on fitness improvements [7].

Delay discounting may be shaped in part by economic scarcity, that is, not enough financial resources to meet a need. Scarcity theory integrates cognitive psychology and economics to explain behaviors among those with not enough resources [20]. Economic (i.e., financial) scarcity may cause someone to focus on immediate resources such as getting food on the table, paying rent, or getting gas into the car, rather than saving for other goals that require prospective thinking. Economic indicators that may induce a scarcity mindset, i.e., an immediate feeling of lack of resources, include household income [21], educational attainment [22], and Medicaid status [23]. Shah and colleagues [24] theorize that scarcity is a mindset in which individuals shift their focus to short-term needs (e.g., borrowing money) rather than longer-term goals (e.g., enrolling in assistance programs) based on immediate resource limitations. These efforts are often counterproductive and may inadvertently reinforce conditions that are associated with scarcity [24]. In a psychobiological model of scarcity and executive function, Kraft and Kraft [25] suggest the relationship between insufficient resources and poor decision-making mechanisms is a result of exposure to harsh environments, which increase stress and inflammation. These biological changes impact neural systems in a way that reinforces decreased reflective self-control, which is associated with steeper discounting rates. When the stress associated with scarcity environments is alleviated, more adaptive decision-making may resume [25]. Through this pathway, lower income is predictive of discounting [26], which may be explained in part as a trade-off of long-term benefits to meet immediate needs [27, 28]. Indeed, low socioeconomic status is related to an increase in food insecurity [29, 30], which is related to weight disparities, or an increased prevalence in youth and adults with low socioeconomic status [29, 31]. Notably, delay discounting moderates the relationship between low income and high food insecurity in adults [32]. In addition, when food availability is unpredictable, and people may experience extended periods of food deprivation, the reinforcing value of food can increase, particularly among parents of children [33–35]. Economic scarcity may also increase a person’s cognitive load in working to create solutions to food scarcity and food insecurity, which can negatively influence decision making by shifting a person's focus on solutions that solve immediate, rather than longer term issues [36]. It is conceivable that an economically scarce environment with increased stress and unpredictable access to food may shape food-making decisions in a way that lends itself to the development and maintenance of an increased weight status via discounting rate. There are many economic factors that are negatively associated with obesity, including household income, food insecurity, and parent educational attainment [37]. It is plausible that understanding the interactions between economic scarcity and delay discounting may provide insight to the mechanisms of these disparities in weight status.

While economic scarcity tends to be stable throughout generations within a family [38], children and parents may experience the effects of economic scarcity differently, due in part to differential experiences based on developmental and neurobiological responses to environmental conditions [39]. If the environments associated with economic scarcity are experienced differently for parents and children, there may be differences in the relationship between scarcity and delay discounting in parents and children.

The associations between factors related to economic scarcity and delay discounting for parents and children within families is unclear. The purpose of this study is to cross-sectionally assess the relationships among indicators of income scarcity and delay discounting in parents and their children with obesity enrolled in a family-based behavioral weight management intervention. Based on previous research [40, 41], it is expected that parents and children will experience scarcity differently, and indicators of economic scarcity will have a greater impact on parents than on children, as parents may attempt to shield their children from the effects of economic scarcity [39, 42].

## Methods

### Participants and methods

This dataset is a subset of the Primary care pediatrics, Learning, Activity, and Nutrition (PLAN) study (NCT02873715) (19/08/2016). The protocol for this study is described elsewhere [43]. Participants were parent- 6–12-year-old child dyads with overweight or obesity. Briefly, enrolled parents had a BMI at or above 25 and target children included a BMI at or above the 85^th^ percentile for their age and sex. Criteria for participation included the ability to read and comprehend English language materials, and for the parent to be the target child’s biological or adoptive parent or legal guardian with whom the child resides at least 50% of the time. Exclusion criteria included any physical or mental disorder, medical treatment, or fasting practices that would prohibit engagement with the intervention or the traffic light eating plan. After screening, baseline assessments were collected, and 452 child-parent dyads were randomized to Family-Based Behavioral Treatment or Usual Care at their primary care provider’s office [43]. The trial was approved by the Institutional Review Board (IRB) at the University at Buffalo (MODCR00006087), with Reliance Agreements across each site. All methods were carried out in accordance with the Declaration of Helsinki. Informed consent was obtained from all subjects and/or their legal guardians; adult consent, parent permission, and child assent were collected prior to participation.

### Measures

#### Demographic variables

At baseline, data were collected on race (Asian, Black, Multiracial, Native, Other, Refused, White), ethnicity (Hispanic/Latino, or not Hispanic/Latino), years of education, family size, household income, and type of parent insurance coverage. Income was split using low category as -1 standard deviation units from the median, high as + 1 standard deviation units from the median, and median income as those within -1 to + 1 standard deviation limits. Insurance coverage was categorized by either parent Private insurance (traditional employer based or purchased), Medicare, Medicaid, Veterans Insurance (Tri insurance or Veterans insurance), Universal Health Care (Canada), Supplementary insurance, or other insurance.

#### Scarcity

Primary indicators of economic scarcity were educational attainment, combined family income [44, 45] and dichotomous coding of insurance for those parents with Medicaid or not [45, 46]. These variables were obtained from the demographic questionnaire completed by participants.

#### Adjusting amount delay discounting task

All participants completed an adjusting amount delay discounting task at the baseline/screening session, and each assessment session [47]. During this task, participants chose between receiving a larger amount of money at a future time point (1 day, 7 days, 30 days, 182 days, 365 days) or a smaller amount of money now (e.g., *“Would you rather have $50 now or $100 in one week?)*. The amount of money offered “now” adjusted based on participant’s prior response. For each participant, indifference points, or the amount of money offered now that was just as appealing to them as $100 at a future time point, were established.

Ordinal area under the curve (AUC^ord^) was used as the measure of delay discounting. In delay discounting paradigms, the construct of discounting is that the value (measured as the indifference point) of a reward can be reduced as the amount of time to receive the award varies; typically, the longer the delay the less the reward can hold its value. Values, or indifference points are calculated for each of the delays, and these are used to calculate the AUC^ord^.

AUC^ord^ ranges from 0 (maximum discounting of the delayed reward) to 1 (no discounting); higher values of AUC^ord^ indicate lower levels of discounting. AUC^ord^ was calculated using the ordinal values for each of the five future time points or delays. This measure was chosen over the traditional AUC measure because it does not give more weight to the contribution of increased delays [48]. It is common in studying delay discounting research to check for non-systematic responding, which can reduce confidence in the quality of the data [49]. The algorithm assesses whether an indifference point was greater than the preceding indifference point by greater than 20% of the larger later reward or less than 10% of the larger later reward. Systematic responses would be those that fall within these limits, while non-systematic responses are those that exceed these limits. Children are more likely to provide non-systematic data than adults [50].

#### Anthropometric measures

Trained study coaches measured child and parent height and weight. Height was measured using a standard stadiometer and protocol that requires calibration and reliability within 0.3 cm. Weight was measured using a medical-grade SR Instrument Portable Scale. Study coaches were trained in weight calibration and protocol to meet the reliability requirement within 0.25 lbs. Parent and child height and weight data were used to calculate adult BMI and child percent over the median BMI. Percent over the median BMI is calculated as [(actual BMI minus BMI at the 50th percentile)/ BMI at the 50th percentile, using age and sex appropriate norms from CDC growth charts. For parents median BMI used sex normalized values at age 20 as proxy for adults [51].

The formula for calculating LMS z-BMI scores based on age and sex specific LMS parameters from CDC growth charts (L = power, M = median, and S = standard deviation) and are calculated as zBMI = ((BMI/M^L^)-1)/L*S) [52].

#### Analytic plan

Analyses were carried out using all available systematic data for parents (*N* = 334, 81.3%) and children (*N* = 237, 57.7%), as well as sensitivity analyses including all data collected from parents and children (*N* = 411). Zero-order Pearson product moment correlations were calculated to examine the association between parent and child delay discounting and continuous demographic variables (age, sex, years of education, income) and point bi-serial correlations for dichotomous/dummy coded variables (sex, minority status, Medicaid status).

Hierarchical linear models were used to assess differences in the relationship between parent and child variables of interest and delay discounting, by testing the interaction of parent/child dummy code * each predictor. Data for children and parents were stacked with the parent and child nested within family, and family member (parent or child) dummy coded. Models used AUC^ord^ as the dependent variable and included each predictor, parent–child dummy code, and the interaction to test for differences in the relationship. Additional sensitivity analysis included covariates of age, sex, and race. For example, the model to test the whether parent/child status moderates the relationship between income and delay discounting is AUC^ord^ = β_0j_ + β_1j_*Income + β_2j_ *Parent/Child dummy code _ij_ + β_3j_* Income*Parent/Child dummy code_ij_ + error _ij_, while the model including covariates is AUC^ord^ = β_0j_ + β_1j_*Income + β_2j_ *Parent/Child dummy code _ij_ + β_3j_*Age _ij_ + β_4j_*Sex _ij_ + β_5j_*Minority _ij_ + β6j* Income*Parent/Child dummy code _ij_ + error ij. Multiple testing was controlled for using the Benjamini-Hochberg False Discovery Rate (FDR) with a rate of 10% across all correlations and regression models [53]. Unadjusted values are presented, with significant effects indicated.

## Results

### Demographics

Delay discounting was completed for 411 parent–child dyads (90.9% of the sample). Of those participating parents, 85.9% were mothers, and 52.1% of participating children were girls. Greater maternal participation in our sample is consistent with greater involvement in pediatric research [54, 55] and in feeding their children [56]. Characteristics of families in which both parents and children participated are presented in Table 1 for the entire sample of families as well as the sample of parents or children who provided systematic data.

**Table 1:** Descriptives of all responders and those with systematic and non-systematic patterns of responding on delay discounting

	All Participants		Systematic Responders		Non-Systematic Responders		Systematic vs Non-Systematic	
	Parent	Child	Parent	Child	Parent	Child	Parent	Child
							P	
N	411	411	334	237	77	174	< 0.0001	0.0019
Sex (%M)	14.1%	47.9%	14.7%	48.1%	11.7%	57.5%	0.498	0.060
Minority (%)^a^	34.6%	32.1%	30.2%	26.9%	46.8%	41.8%	0.001	0.002
Mean % over median BMI (SD)	65.8 (34.3)	59.6 (27.3)	65.6 (34.4)	58.9 (25.1)	66.6 (33.9)	60.6 (30.3)	0.815	0.526
Mean Age (SD)	41.4 (7.3)	9.4 (1.8)	42.4 (7.3)	9.7 (1.8)	40.0 (7.2)	9.1 (1.8)	0.890	0.002
AUC^ord^ (SD)	.734 (0.237)	.537 (0.282)	.762 (0.213)	.600 (0.306)	.696 (0.263)	.451 (0.220)	< 0.001	< 0.001
Mean zBMI (SD)		2.2 (0.4)		2.2 (0.3)		2.2 (0.4)		0.520
Mean BMI (SD)	37.1 (7.9)		37.0 (7.9)		37.2 (7.7)		0.845	
Medicaid (%)	20.7%		16.5%		26.4%		0.014	
Mean years of education (SD)	15.0 (2.2)		15.3 (2.1)		14.7 (2.3)		0.004	
Mean total household income (SD)	$84,459 ($55,134)		$92,553 ($55,117)		$73,401 ($53,360)		< 0.001	
Mean family size (SD)	4.5 (1.2)		4.4 (1.2)		4.6 (1.2)		0.289	

^a^Minority results are in relationship to presence in minoritized groups that are non-White and non-Hispanic or Latino

### Differences in systematic and non-systematic responders

Families with parents or children with non-systematic data had less educational attainment (*p* = 0.004), lower income (*p* < 0.001), greater percentage of minorities (*p* = 0.002), greater percentage on Medicaid (*p* = 0.014), and their children were younger (*p* = 0.002). Both children (*p* < 0.001) and parents (*p* < 0.001) who were non-systematic responders showed lower AUC (*p* < 0.001).

Analysis of systematic responders showed parents with higher BMI and children with higher percent over median BMI and membership in a minoritized group were greater discounters, as were parents who received Medicaid, had lower income or educational status, were in a minoritized group and were parents in larger families.

### Parent vs child differences in discounting relationships with predictors, all data (systematic and non-systematic)

When all data are considered, the same parental predictors of delay discounting were observed, with the exception of BMI or percent over median BMI. These findings demonstrate no differences between systematic and non-systematic responsive adults in regard to income and minority status. The only predictor of child delay discounting was presence in a minoritized group. Parents with lower income, less education and presence in a minoritized group had significantly greater discounting than their children. Sensitivity analyses replicated these findings when including covariates of age, sex, and minority status.

### Relationships of predictors with delay discounting

Predictors of parent and child delay discounting for all families who completed the delay discounting tasks (*n* = 411), and for only those showing systematic responding (*n* = 334 parents and 237 children) data are presented in Table 2.

**Table 2:** Correlations of predictors with delay discounting (BAUC^ord^) for all data and systematic responder data^a^

Predictor	All Dyads			Systematic Responders		
	Parent	Child	Parent vs Child	Parent	Child	Parent vs Child
	r (p)	r (p)	p	r (p)	r (p)	p
BMI	-0.08 (0.10)	-0.09 (0.06)	0.36	-0.112* (0.035)	-0.17* (0.009)	0.056
% over BMI	-0.08 (0.09)	-0.9 (0.07)	0.58	-0.12* (0.028)	-0.156* (0.017)	0.15
Medicaid	-0.15* (0.002)	-0.003 (0.95)	0.056	-0.15* (0.006)	0.06 (0.35)	0.021*
Education	0.24* (< 0.001)	0.03 (0.51)	0.008*	0.21* (0.001)	0.02 (0.82)	0.059
Income	0.28* (< 0.001)	0.08 (0.10)	0.016*	0.25* (< 0.001)	0.01 (0.84)	0.019*
Minority^b^	0.30* (< 0.001)	0.11* (0.02)	0.03*	0.28* (< 0.001)	0.08 (0.24)	0.089
Family Size	-0.11* (0.034)	-0.05 (0.37)	0.50	-0.13* (0.018)	-0.03 (0.65)	0.38
Age	0.06 (0.22)	-0.06 (0.21)	0.11	0.09 (0.09)	-0.14* (0.03)	0.005*
Sex^b^	0.05 (0.27)	-0.04 (0.50)	0.18	0.10 (0.07)	-0.06 (0.32)	0.054

^*^Significant controlling for 54 tests with 10% False Detection Rate using Benjamini–Hochberg procedure

^a^Differences in parents vs. children were assessed based the predictor * parent/child dummy code term from each hierarchical linear model

^b^Minority results are in relationship to presence in minoritized groups that are non-White and non-Hispanic or Latino, while sex is in reference to being male. Male is coded as 1, female is coded as 2

### Parent vs child differences in discounting relationships with predictors, systematic-data only

**Fig. 1:**
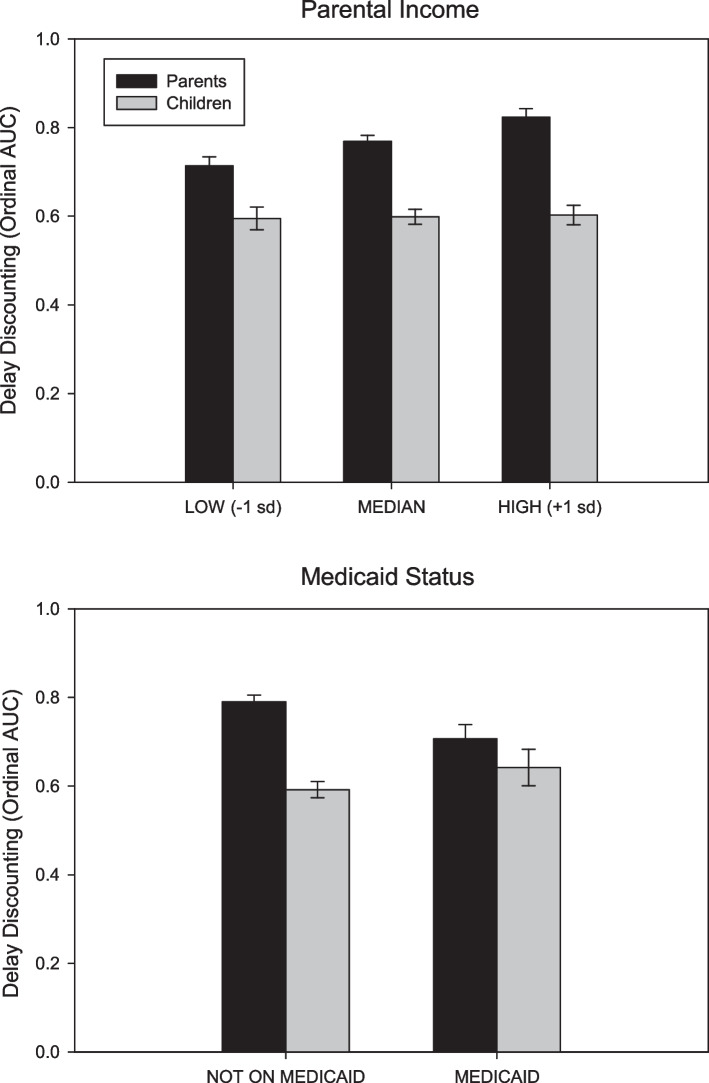
Model estimates of delay discounting (AUCord) based on income (Top) and Medicaid status (Bottom)

Mixed model comparisons of parent and child relationships showed discounting was greater for parents with lower parental income (Fig. 1, top graph) and who received Medicare (Fig. 1, bottom graph) and who belonged to a minoritized racial/ethnic group.

## Discussion

Consistent with other research, we found that indicators of economic scarcity, including lower income, education status, and Medicaid status, were related to discounting of the future in adults who had overweight or obesity and volunteered for the PLAN study [11, 16, 57, 58], confirming our main hypothesis. These results show that adults with overweight /obesity who have scarce resources may have differences in decision making that favor immediate, rather than delayed consequences. Among this group, those with limited resources are shaped to focus on immediate needs, not on future goals. In addition, poverty and low-income/low socio-economic status in adults are consistently linked with stressors that can increase cognitive load, worry, depression, anxiety, and disproportionately increase rates of mental illness among adults [59–61]. The employment of these cognitive resources to focus on immediate needs, in addition to added stress from social stigma associated with SES, may impair decision-making processes in a way that exacerbates scarce conditions [62].

While the relationship between indicators of scarcity and delay discounting is clear for parents in our study, these factors did not have the same impact on delay discounting for children with overweight and obesity in these families. One possibility may be that parents and children experience the same environmental context differently, as research has found children to be significantly less likely to be reported as experiencing food insecurity compared with adults living in their same household [63]. Mothers may shield their children from the effects of food security by reducing their own food intake in order to make this limited resource available for the children, known as “maternal deprivation” [64]. Another possibility is a differential response to the stress of living in a lower resourced environment for parents and children. Crandall and colleagues [65] found food insecurity to affect the stress response (i.e., cortisol levels) during simulated acute financial losses in parents, but these relationships among food insecurity and stress response were not found among their child or adolescent offspring. Among adults, those who experience economic scarcity also experience greater distress intensity in their daily lives [66]. As children may not yet experience the compound effects of chronic stress and financial scarcity [67], these factors may not have the same effect on executive functions, including delay discounting, as has been observed in adults [62]. It is important not to disregard the potential effects of childhood poverty, which may be associated with increased levels of stress and executive function deficits into adulthood [68], and early experiences of stress and economic scarcity may be a pathway for steep discounting in adulthood. Parents may attenuate the negative impacts of scarcity if they are resilient [42] and in good mental health [69], which may allow them to make resources for healthy lifestyle more available for their children than themselves [70, 71], and by providing emotional or affective support [72].

For adults, policy makers may be able to bolster health related interventions and outcomes that are bidirectionally associated with delay discounting by implementing programs and strategies that reduce the effects of scarcity, for example providing greater access to resources to meet financial and medical needs adequately. Similarly, adults living in these conditions may mitigate the effects of scarcity by engaging in practices that can improve delay discounting rates [25, 73]. There is support to show that delay discounting rate is a *trait-like* state (e.g., steep discounting for monetary rewards) and when modified, improvements can be evident across other types of outcomes (e.g., food intake) [74]. Episodic future thinking (EFT) is an effective intervention to improve delay discounting rate. EFT employs vivid engagement in positive thinking about future events in order to choose a larger, later reward. EFT has been shown to improve discounting rate among people with prediabetes and who are seeking weight loss [75]. Adults may also consider financial planning, which has demonstrated benefits of improving valuation of long-term rewards [76] as well as financial benefits, particularly for those who experience economic scarcity [32].

The current study has a number of strengths including its large and diverse sample size but is limited by a restricted BMI range and cross-sectional data that cannot attribute cause or effect. Since people with obesity are more likely to discount the future [3, 6], they may be more susceptible to effects of economic scarcity, and these factors may increase discounting in an additive or synergistic manner. Acknowledging these considerations, we believe the current findings shed new light on the relationships among indicators of economic scarcity and socioeconomic status and delay discounting among families with parents and children with obesity. Further research is warranted to understand how parents protect their children from the cognitive and behavioral effects of economic scarcity.

It is important to identify and describe *who* is at greatest risk for experiencing the compound effects of stress and scarcity, and how this will interact with delay discounting rate and related health outcomes. Research is needed to understand interventions that can promote the consideration of larger, future rewards, versus smaller, sooner rewards for those who experience economic scarcity and if these interventions can improve health-related choices and address weight disparities. Prospective research may benefit from understanding the pathways between experiences of scarcity and stress in childhood, to discounting rate development and decision-making processes in adulthood. A final pathway to consider for exploration is the association between education level and potential lower health literacy as it relates to scarcity and delay discounting rate.

Adults with overweight or obesity who experience scarcity or who have lower educational attainment are more likely to have increased rates of delay discounting, while these same effects are not yet present in their children. Our findings support scarcity theory that suggests individuals who lack necessary resources are shaped to develop a cognitive style to meet immediate versus long-term needs, although it is also possible that those individuals who discount the future are more likely to experience economic scarcity than individuals with a more prospective mindset. These findings have implications for the assessment and specific targeted interventions for families who experience economic scarcity and who seek behavioral health and obesity treatment. The significant effects of indicators of scarcity on delay discounting, a trans-diagnostic marker [4, 5] indicate a need to identify behavioral and economic solutions to reduce scarcity and improve executive function in those experiencing these conditions.

## Data Availability

The datasets generated and analyzed during the current study will be placed in clinicaltrials.gov data repository. Point Of Contact: Leonard H. Epstein, lehenet@buffalo.edu.
